# Land Subsidence in Qingdao, China, from 2017 to 2020 Based on PS-InSAR

**DOI:** 10.3390/ijerph19084913

**Published:** 2022-04-18

**Authors:** Mengwei Li, Xuedong Zhang, Zechao Bai, Haoyun Xie, Bo Chen

**Affiliations:** 1 School of Geomatics and Urban Spatial Informatics, Beijing University of Civil Engineering and Architecture, Beijing 102627, China; 2108160120004@stu.bucea.edu.cn (M.L.); 2108160321010@stu.bucea.edu.cn (H.X.); 2108160321001@stu.bucea.edu.cn (B.C.); 2 School of Electrical and Control Engineering, North China University of Technology, Beijing 100144, China

**Keywords:** Qingdao, land subsidence, PS-InSAR, subway construction

## Abstract

Land subsidence is a global geological disaster that seriously affects the safety of surface and underground buildings/structures and even leads to loss of life and property. The large-scale and continuous long-time coverage of Interferometric Synthetic Aperture Radar (InSAR) time series analysis techniques provide data and a basis for the development of methods for the investigation and evolution mechanism study of regional land subsidence. Based on the 108 SAR data of Sentinel-1 from April 2017 to December 2020, this study used Persistent Scatterer InSAR (PS-InSAR) technology to monitor the land subsidence in Qingdao. In addition, detailed analysis and discussion of land subsidence combined with the local land types and subway construction were carried out. From the entire area to the local scale, the deformation analysis was carried out in the two dimensions of time and space. The results reveal that the rate of surface deformation in Qingdao from 2017 to 2020 was mainly −34.48 to 5.77 mm/a and that the cumulative deformation was mainly −126.10 to 30.18 mm. The subsidence areas were mainly distributed in coastal areas (along the coasts of Jiaozhou Bay and the Yellow Sea) and inland areas (northeast Laixi City and central Pingdu City). In addition, it was found that obvious land subsidence occurred near the Health Center Station of Metro Line 8, a logistics company in Qingdao, and near several high-rise residential areas and business office buildings. It is necessary for the relevant departments to take timely action to prevent and mitigate subsidence-related disasters in these areas.

## 1. Introduction

Urban land subsidence is defined as local downward movement that lowers the elevation of the crust’s surface due to the consolidation and compression of loose underground strata under the influence of human engineering activities. With the acceleration of urbanization, an increasing number of cities have experienced land subsidence. Some areas have even become the areas hardest hit by subsidence, causing huge economic losses, affecting the safe development of cities, and threatening people’s lives and health [1,2,3]. Land subsidence was first recorded in Mexico City in 1891. In 1921, the phenomenon of land subsidence was also discovered in Shanghai, China [4]. Soon after, countries gradually paid more attention to land subsidence-related disaster, which is now listed as a worldwide research problem, and corresponding measures have been taken to avoid subsidence and its related disasters.

In recent years, with the rapid development of Synthetic Aperture Radar Interferometry (InSAR), a new remote sensing monitoring method has become available for researching and monitoring urban land subsidence. InSAR uses two SAR images taken at the same position but at different times to extract the displacement of the target in the satellite’s line of sight (LOS) after processing. Therefore, it can be used to monitor deformation of the Earth’s surface, such as volcanoes, earthquakes, debris flows, landslides, glacier movement, and land subsidence. In 1989, Gabriel et al. [5] first proposed Differential InSAR (D-InSAR) with centimeter-level accuracy, which has since been widely used to monitor surface deformation. However, due to atmospheric effects, decoherence, and other factors, D-InSAR has difficulty meeting the needs of monitoring urban land subsidence characterized by millimeter-scale deformation. With the accumulation and quality of SAR data, many scholars have begun to study the use of long time series SAR datasets to improve the accuracy of surface deformation inversions. In 1998, Sandwell and Price [6] proposed the stacking algorithm for improving the accuracy of deformation monitoring. In 2000, Ferretti et al. [7] of the Politecnico di Milano proposed Persistent Scatterer InSAR (PS-InSAR) and studied the land subsidence phenomenon in Pomona, Italy, based on European Remote Sensing (ERS) satellite images. In 2002, Berardino et al. [8] proposed Small Baseline Subset InSAR (SBAS-InSAR) and conducted deformation monitoring of the Campi Flegrei volcano and the Italian City of Naples using European remote sensing satellite data from 1992 to 2000. This technique reduces the spatial decorrelation by selecting a smaller baseline, thereby increasing the number of coherent pixels in the differential interferogram and improving the spatial resolution of the deformation monitoring. Based on the above concepts, many new InSAR techniques have been developed in recent years [9,10,11,12]. The new algorithms mainly include Interferometric Point Target Analysis (IPTA) for improved robustness [13], the Stanford Method for Persistent Scatterers (StaMPS) algorithm for monitoring low-coherence regions [14], the SqueeSAR joint Distributed Scatterer (DS) and Persistent Scatterer (PS) point solution [15], and the Coherent Target (CT) based on coherent coefficient selection method [16]. These algorithms have effectively promoted the rapid development of InSAR technology.

Based on the development of InSAR technology, many scholars have conducted related research on urban land subsidence using time series InSAR [17,18,19]. Based on Advanced Land Observing Satellite (ALOS) Phased Array L-band Synthetic Aperture Radar (PALSAR) data collected from January 2007 to March 2011, Chen et al. [20] improved the InSAR processing process to obtain the deformation in a vegetated groundwater basin in the San Luis Valley in Colorado, USA, and revealed the storage characteristics of the aquifer in the region using InSAR well data. Lu et al. [21] used ENVISAT Advanced Synthetic Aperture Radar (ASAR) data collected from 2004 to 2010 to study the spatial and temporal distributions of the land deformation in Changzhou, China, using SBAS technology. Combined with leveling data, they analyzed the subsidence caused by excessive groundwater exploitation in detail. Hu et al. [22] used ENVISAT ASAR data collected from 2004 to 2010 and Sentinel-1A data collected from 2015 to 2016 to obtain the deformation field in Salt Lake City, UT, USA, using multi-temporal InSAR. Combined with hydrological data, the seasonality of the long-term deformation was revealed, and the different reasons for the subsidence were analyzed. Dong et al. [23] used Radarsat-2 data collected from 2011 to 2013 to obtain the vertical land deformation in Shanghai, China, using SBAS technology and used the ascending and descending data collected from April to August 2008 to obtain the vertical and horizontal land deformation in Shanghai, China, using Multidimensional SBAS (MSBAS) technology. Cigan et al. [24] inverted the three-dimensional deformation field in Mexico City based on Sentinel-1 data collected from 2014 to 2020 using D-InSAR and SBAS technology, analyzed the causes of the different subsidence areas, and carried out a risk assessment. He et al. [25] combined Time Series InSAR (TS-InSAR) and Light Detection and Ranging (LiDAR) technology to monitor the ground and buildings in Shenzhen, China, analyzed the causes of the subsidence, and provided a scientific reference for urban planning.

As one of the important central cities along the coast of China, Qingdao is not only an international port but also a center of the tourism industry and promotes economic development. In recent years, Qingdao’s urbanization has developed rapidly, and an increasing number of subways and high-rise buildings have been constructed. In order to prevent land subsidence from endangering peoples’ lives and property, it is necessary to further strengthen the monitoring and prevention of large-scale urban land subsidence [26]. Therefore, in this study, 108 Sentinel-1A radar satellite images acquired from April 2017 to December 2020 were used to monitor and analyze the land subsidence throughout the entire city of Qingdao using PS-InSAR. The total monitoring area was 11,293 km^2^, and 334,194 PS points were extracted. Compared with previous studies, more comprehensive land subsidence information was obtained over a longer monitoring time period. The large-scale regional InSAR deformation monitoring results provide an important reference and scientific basis for the investigation of land subsidence in Qingdao. In addition, the detailed analysis of the area experiencing excessive subsidence provides auxiliary data for the relevant departments to implement corresponding measures to avoid serious collapse accidents.

## 2. Study Area and Data

### 2.1. Study Area

**Figure 1:**
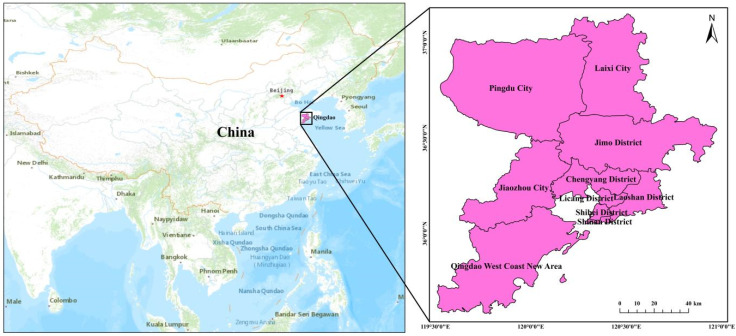
Geographical location of the study area.

The study area in this study was Qingdao City, Shandong Province, China (Figure 1). Qingdao is located in the southeastern part of the Shandong Peninsula (119°30′00′′–121°00′00′′ E, 35°35′00′′–37°09′00′′ N). It covers an area of 11,293 km^2^. Mountains account for 15.5% of the total area of the city, hills account for 2.1% of the total area of the city, plains account for 37.7% of the total area of the city, and depressions account for 21.7% of the total area of the city. Qingdao, which is located on the Laoshan granite, has excellent building foundation conditions. The structure is dominated by fault structures, and the area is dominated by fault-block uplift with a relatively stable integrity. The amount of uplift is generally small. As a coastal hilly city, Qingdao’s terrain is high in the east and low in the west, uplifted on both the north and south sides, and concave in the middle. Qingdao is located in the northern temperate monsoon area and has a temperate monsoon climate. It also has obvious indigenous marine climate characteristics. The air is moist, the rainfall is abundant, the temperature is moderate, and the four seasons are distinct [27].

### 2.2. Data

In this study, a total of 108 SAR images acquired by Sentinel-1A during the European Space Agency Copernican Plan from April 2017 to December 2020 were selected. The properties of the dataset are presented in Table 1. The orbital accuracy of the Sentinel-1 images was corrected using the satellite’s precision orbit data. The effect of the topographic phase was eliminated using 90 m resolution Shuttle Radar Topography Mission (SRTM) Digital Elevation Model (DEM) data provided by the National Aeronautics and Space Administration (NASA).

## 3. PS-InSAR

PS-InSAR refers to Permanent Scatterer Synthetic Aperture Radar Interferometry. The principle of PS-InSAR is to use N scene SAR images of the same study area. One scene image was selected as the master image, and the N − 1 scene images were selected as the slave images. PS candidate points were selected based on the Amplitude Dispersion Index (ADI). The phase dispersion *σ_v_* can be approximated using the dispersion of the amplitude, as follows:

(1) $$\sigma_{v}≅\frac{\sigma_{n_{R}}}{g}≅\frac{\sigma_{A}}{m_{A}}=D_{A}$$

where *σ_nR_* is the standard deviation of the complex real part, *g* is the complex observation value, *m_A_* and *σ_A_* are the mean and standard deviation of the amplitude, respectively, and *D_A_* is the deviation of the amplitude. N − 1 differential interferograms are obtained by subtracting the phase value of the PS points from each scene slave image. The information for each differential interferogram is as follows:

(2) $$\Phi =\Phi_{\mathrm{def}}+\Phi_{\varepsilon }+\Phi_{\mathrm{atm}}+\Phi_{\mathrm{orb}}+\Phi_{\mathrm{noi}}$$

where *Φ* is the interferometric phase, *Φ_def_* is the deformation phase in the LOS direction, *Φ_ε_* is the terrain phase caused by the DEM error, *Φ_atm_* is the atmospheric delay phase, *Φ_orb_* is the orbital deviation phase, and *Φ_noi_* is the error component caused by the thermal noise and registration process. The phase of the atmospheric error, the DEM residual, the surface deformation, and the orbital error are estimated using a network composed of PS points and the temporal and spatial characteristics of each phase component. Then, the deformation information is estimated [28,29,30].

**Figure 2:**
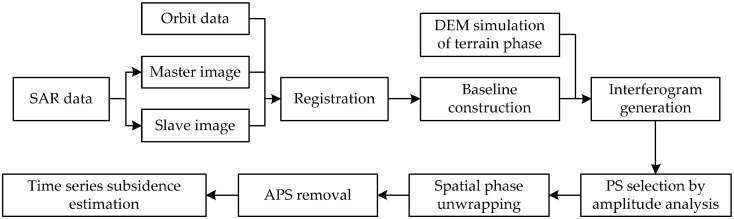
The flowchart for PS-InSAR.

Because PS points are less affected by temporal and spatial incoherence and their phase information and amplitude information are stable, they can maintain stable scattering characteristics in a long time series of images and dominate the radar signal characteristics of the image resolution units where they are located. Thus, these resolution units maintain a high coherence over a long time period. Then, high-precision deformation information can be obtained after removing the Atmospheric Phase Screen (APS) and noise phase. The measurement accuracy can reach the millimeter level [7]. The main workflow of the PS-InSAR processing is shown in Figure 2.

## 4. Results and Analysis

### 4.1. Overall Analysis

**Figure 3:**
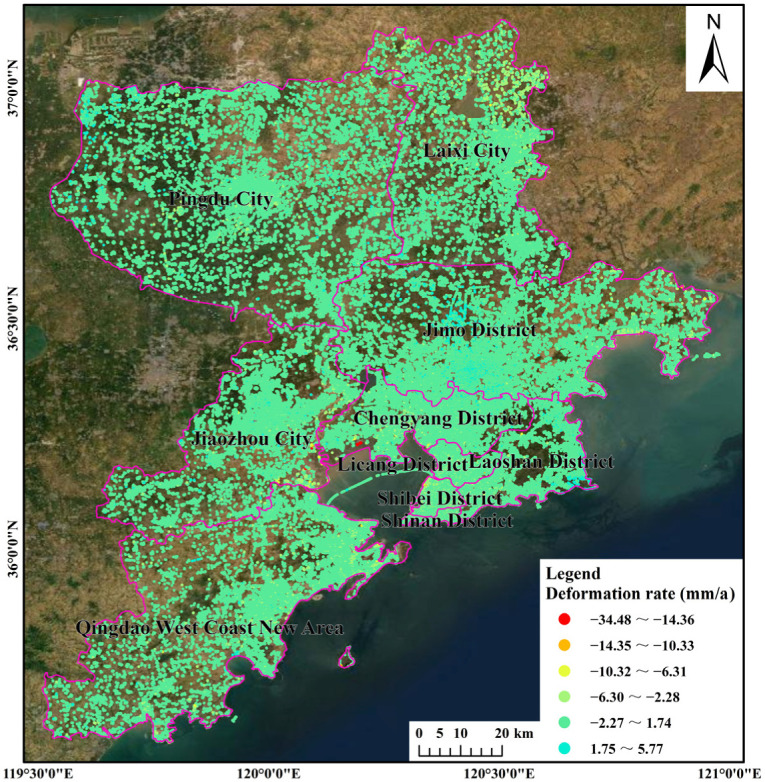
Land subsidence rate in Qingdao from 2017 to 2020.

The land subsidence rate in Qingdao was obtained through inversion of Sentinel-1A images (Figure 3). The coordinates of the reference point are (36.371189°, 119.732767°). There were a total of 334,194 PS points, and the density of the PS points was about 29.59 per square kilometer. It can be seen from Figure 3 that the overall ground situation in Qingdao was relatively stable from 2017 to 2020, the surface deformation rate was mainly –34.48 to 5.77 mm/a, and the cumulative deformation was mainly −126.10 to 30.18 mm. The obvious subsidence areas were mainly concentrated along the coasts of Jiaozhou Bay and the Yellow Sea. The subsidence was relatively scattered and was sporadically distributed. The general subsidence areas were mainly concentrated in the northern part of Laixi City and the central part of Pingdu City, exhibiting a state of slow subsidence and a concentrated circular distribution. In addition, there was a slight ground rebound phenomenon in the central part of the Jimo District, the eastern part of the Laoshan District, and the western part of Pingdu City.

**Figure 4:**
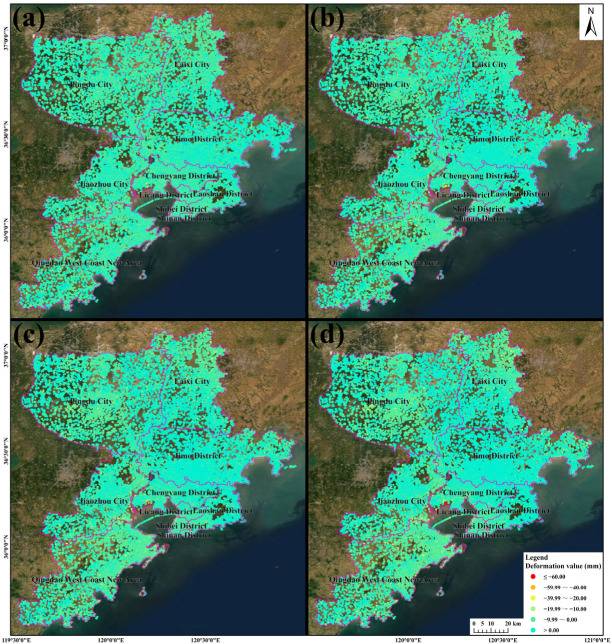
Annual variation in the land cumulative deformation in Qingdao: (**a**) April–December 2017; (**b**) April 2017–December 2018; (**c**) April 2017–December 2019; and (**d**) April 2017–December 2020.

**Figure 5:**
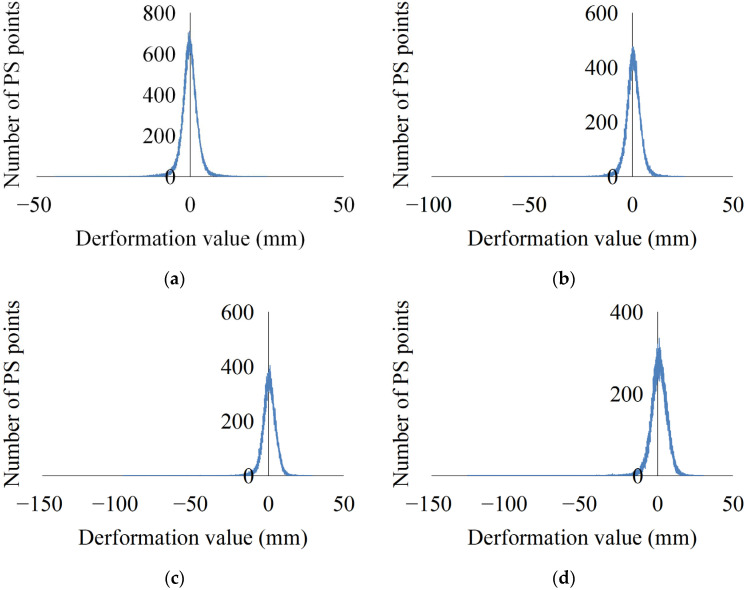
Statistics of the deformation values: (**a**) April–December 2017; (**b**) April 2017–December 2018; (**c**) April 2017–December 2019; and (**d**) April 2017–December 2020.

In this study, 4 April 2017 was taken as the initial time for the calculation of the deformation in order to calculate the annual cumulative deformation (Figure 4). In order to quantitatively reflect the deformation results, the deformation corresponding to the above time periods was counted (Figure 5). As can be seen from Figure 4 and Figure 5, as of December 2017, the deformation was mainly −8.00 to 8.00 mm. The land subsidence along Jiaozhou Bay in the Chengyang District was more than 40.00 mm, and the maximum subsidence was 43.78 mm. The land subsidence along the northeast coast of the Yellow Sea in the Qingdao West Coast New Area exceeded 20.00 mm. As of December 2018, the deformation was mainly −11.00 to 11.00 mm. The land subsidence along Jiaozhou Bay in the Chengyang District and along the northeast coast of the Yellow Sea in the Qingdao West Coast New Area exceeded 60.00 mm, and the maximum subsidence was 63.66 mm. As of December 2019, the deformation was mainly −12.00 to 12.00 mm. The land subsidence along Jiaozhou Bay in the Chengyang District and along the northeast coast of the Yellow Sea in the Qingdao West Coast New Area exceeded 90.00 mm, and the maximum subsidence reached 96.37 mm. As of December 2020, the deformation was mainly −12.00 to 12.00 mm. At this time, the deformation had stabilized, and it was basically consistent with the overall deformation as of December 2019. The land subsidence along Jiaozhou Bay in the Chengyang District and along the northeast coast of the Yellow Sea in the Qingdao West Coast New Area exceeded 120.00 mm, and the maximum subsidence reached 126.10 mm. It can be seen from Figure 5 that the statistical values of the annual deformation accumulation basically exhibit a normal distribution, but the number of negative areas increases gradually, and the values decrease gradually. The above phenomenon shows that Qingdao is in a stable state of deformation overall, but the subsidence in small local areas is increasing year by year, which will lead to increasing damage related to the land subsidence.

**Figure 6:**
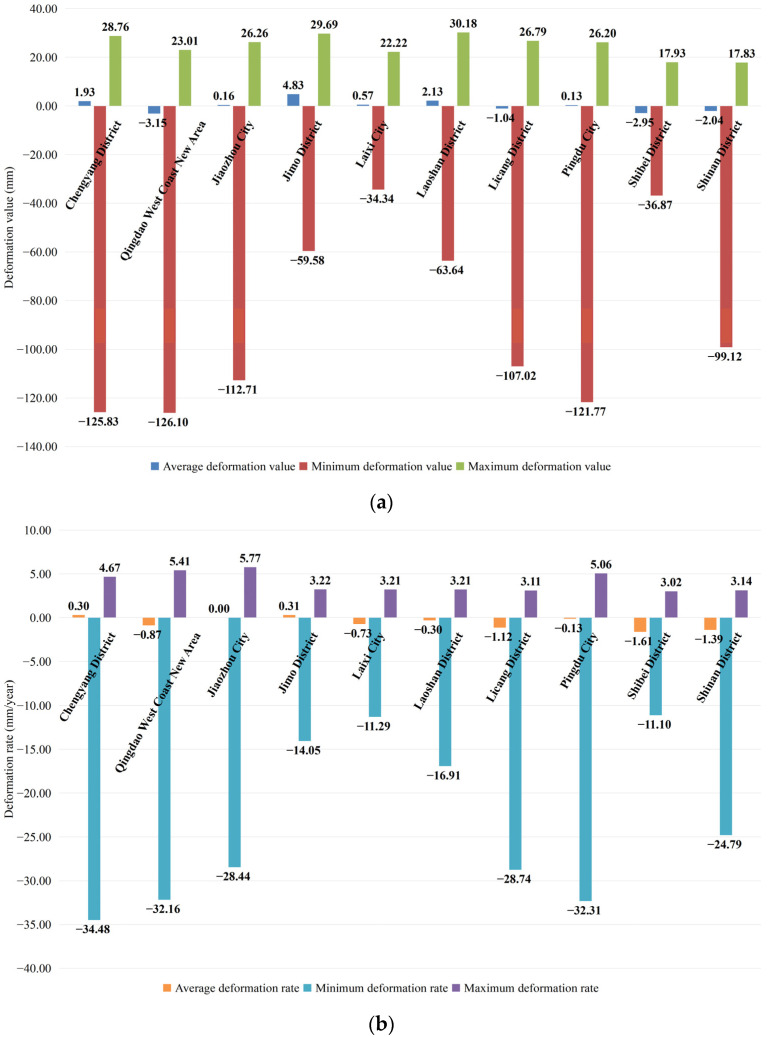
Statistical figures of the deformation rates and cumulative deformation values of the districts/cities in Qingdao: (**a**) deformation rate and (**b**) deformation value.

From the perspective of the spatial distribution, the subsidence rate and the subsidence value in each district/city in Qingdao were extracted and counted in this study (Figure 6). It can be seen from Figure 6 that the overall average deformation rate in each district/city was small, within 2.00 mm/a. The maximum subsidence rate was −34.48 mm/a in the Chengyang District, and the maximum uplift rate was 5.77 mm/a in Jiaozhou City. The overall average deformation in each district/city was small, within 3.00 mm. The maximum subsidence was −126.10 mm in the Qingdao West Coast New Area, and the maximum uplift was 30.18 mm in the Laoshan District. The small average deformation rate and average deformation value indicate that the land deformation throughout the entire area in each district/city tended to be stable, while the much larger subsidence rates and subsidence values indicate that there were areas where the local subsidence was serious. The areas in which the subsidence rate exceeded 20.00 mm/a included the Chengyang District, the Qingdao West Coast New Area, Jiaozhou City, the Licang District, Pingdu City, and the Shinan District. The areas with subsidence values exceeding 60.00 mm included the Chengyang District, the Qingdao West Coast New Area, Jiaozhou City, the Laoshan District, the Licang District, Pingdu City, and the Shinan District. Excessive subsidence rates and subsidence values will threaten the safety of the ground surface. Therefore, in the development of Qingdao, it is necessary to pay attention to the subsidence development trend in the above areas and to implement effective measures in a timely manner to control the subsidence and prevent related disasters.

**Figure 7:**
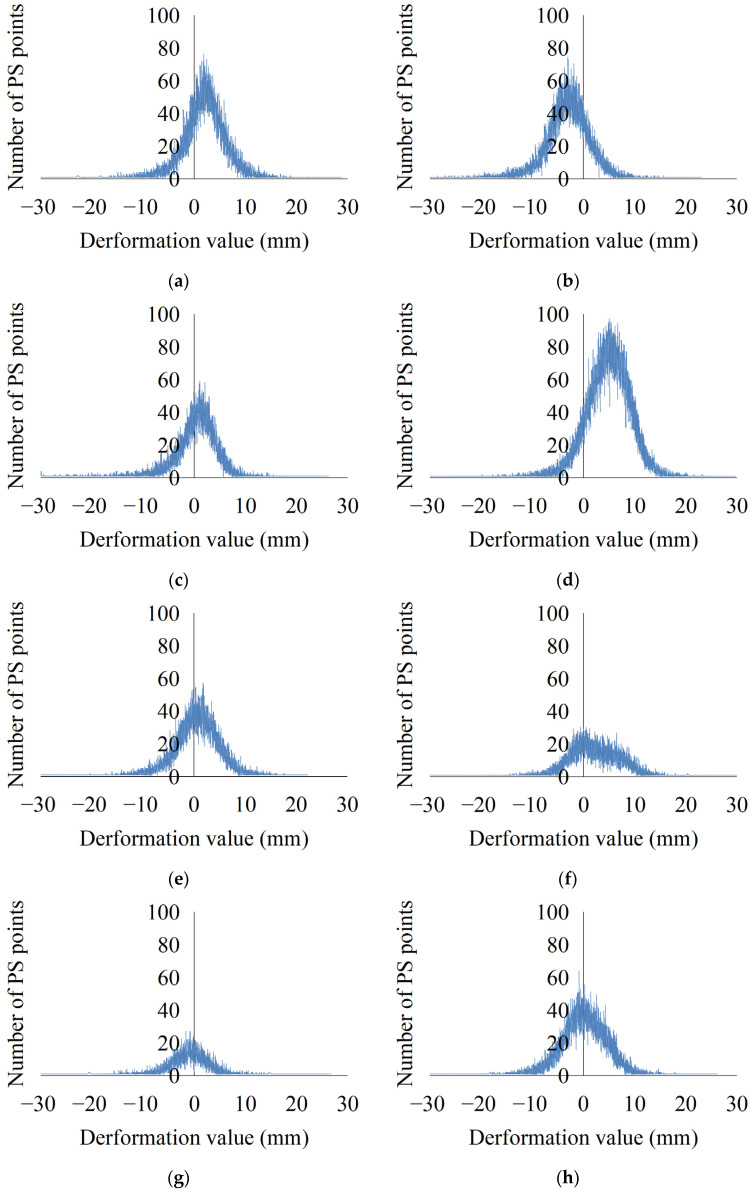

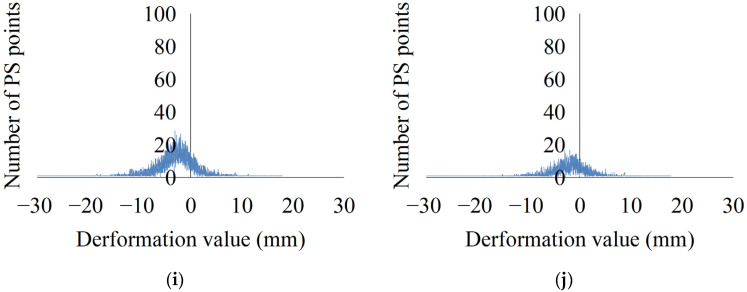
Statistics of the deformation values in the districts/cities in Qingdao. (**a**) Chengyang District; (**b**) Qingdao West Coast New Area; (**c**) Jiaozhou City; (**d**) Jimo District; (**e**) Laixi City; (**f**) Laoshan District; (**g**) Licang District; (**h**) Pingdu City; (**i**) Shibei District; and (**j**) Shinan District.

In order to more comprehensively reflect the land deformation phenomenon in Qingdao, the number of PS points in each district/city was also counted in this study (Figure 7). The results show that the most obvious subsidence area was located in the Qingdao West Coast New Area, where the subsidence was mainly −8.00 to 0.00 mm. The most obvious uplift area was in the Jimo District, where the uplift was mainly 0.00 to 10.00 mm. The deformation of other districts/cities basically conformed to the normal distribution, indicating that the land deformation was relatively stable in most areas. In general, the land deformation in all of the districts/cities in Qingdao was relatively stable, but there was much larger subsidence in some local areas. Therefore, it is necessary to pay attention to these severe subsidence areas, to analyze the causes, and to control and manage them.

### 4.2. Local Analysis

According to the analysis of the overall deformation in Qingdao, the subsidence areas mainly included coastal areas (the coasts of Jiaozhou Bay and the Yellow Sea) and inland areas (northeastern Laixi City and central Pingdu City). Based on the results of the PS-InSAR inversion and the actual conditions in the different areas, the typical areas were analyzed in detail to explore the causes of the deformation.

The maximum subsidence rate along the coast of Jiaozhou Bay reached 34.48 mm/a from 2017 to 2020, and this was the location of the maximum subsidence rate in Qingdao. The subsidence rate along the coast was mainly around 10.00 mm/a, but the deformation exceeded 20.00 mm/a at some deformation points. The overall subsidence along the coast of Jiaozhou Bay was typical in Qingdao, including the Qingdao West Coast New District, Jiaozhou City, the Chengyang District, the Licang District, the Shibei District, and the Shinan District. The maximum subsidence rate along the coast of the Yellow Sea reached 11.90 mm/a from 2017 to 2020. The subsidence area was relatively concentrated. Compared with the subsidence along the coast of Jiaozhou Bay, the deformation along the coast of the Yellow Sea was milder.

**Figure 8:**
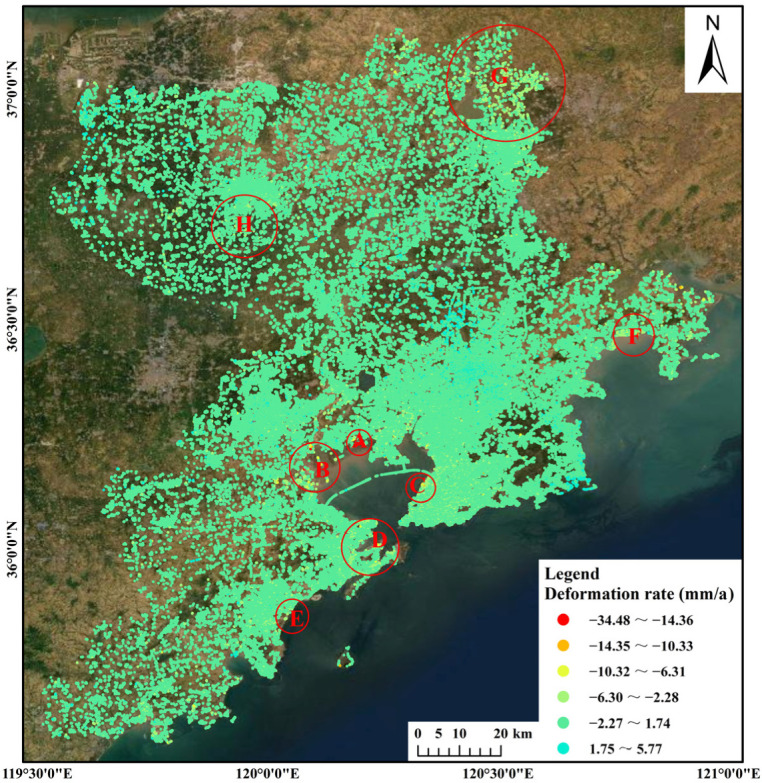
Distribution of subsidence areas.

**Figure 9:**
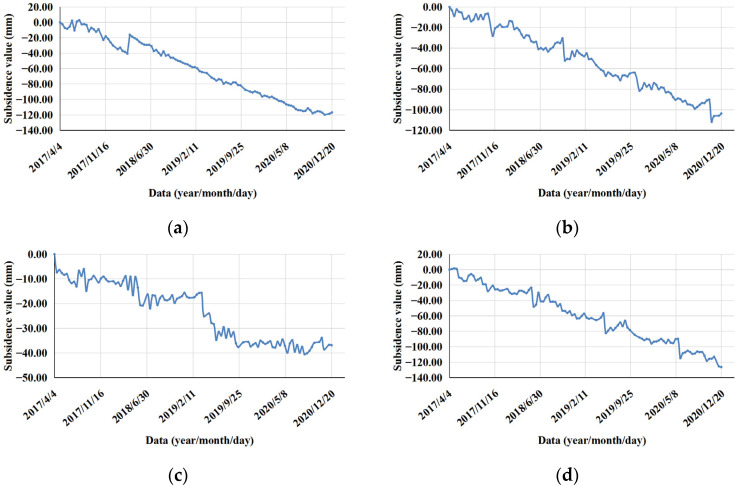

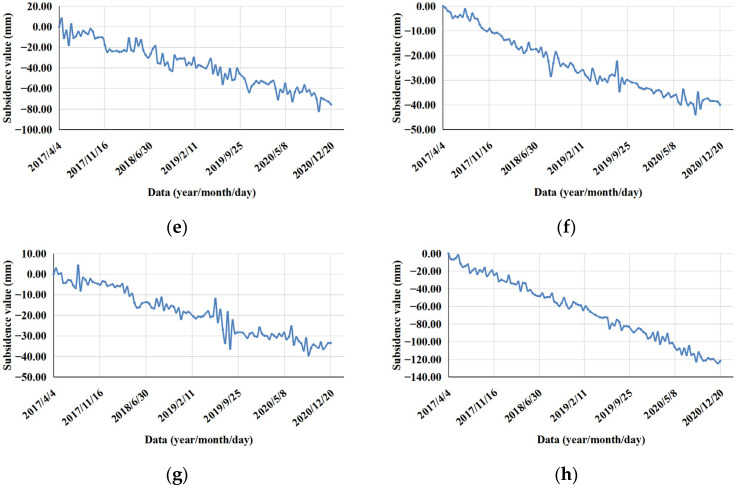
The deformation time curve of the maximum subsidence point in each area: (**a**) Area A; (**b**) Area B; (**c**) Area C; (**d**) Area D; (**e**) Area E; (**f**) Area F; (**g**) Area G; and (**h**) Area H.

In order to further analyze the deformation, the subsidence area in Qingdao was divided into different areas, and the distribution of these areas is shown in Figure 8, including areas A–H. The deformation time curve of the maximum subsidence point in each area is shown in Figure 9.

Combined with the geographic location information, the above local typical subsidence areas were summarized into three categories for discussion and analysis [31]: Area A (area along the subway), Area B (area containing a large logistics company), and Areas C and E (areas containing high-rise buildings). In addition, brief statistics and analyses of the other subsidence areas were carried out.

Area A: This area was roughly located along the northern coast of Jiaozhou Bay. The average subsidence rate was 25.05 mm/a, and the cumulative subsidence was 96.56 mm. Most of the PS points with obvious subsidence were located in the buildings on the south side of the Health Center Station of Metro Line 8. With the development of urbanization, the subway construction in Qingdao has gradually increased. The subway construction and operation have led to land subsidence along the line, which poses a threat to the safety of buildings/structures. Therefore, the subway as one of the causes of subsidence should be focused on. According to the relevant information about the subway construction and operation, the Health Center Station was completed on 19 August 2018, and Metro Line 8 (Jiaozhou North Station-Qingdao North Station) was put into operation on 24 December 2020.

**Figure 10:**
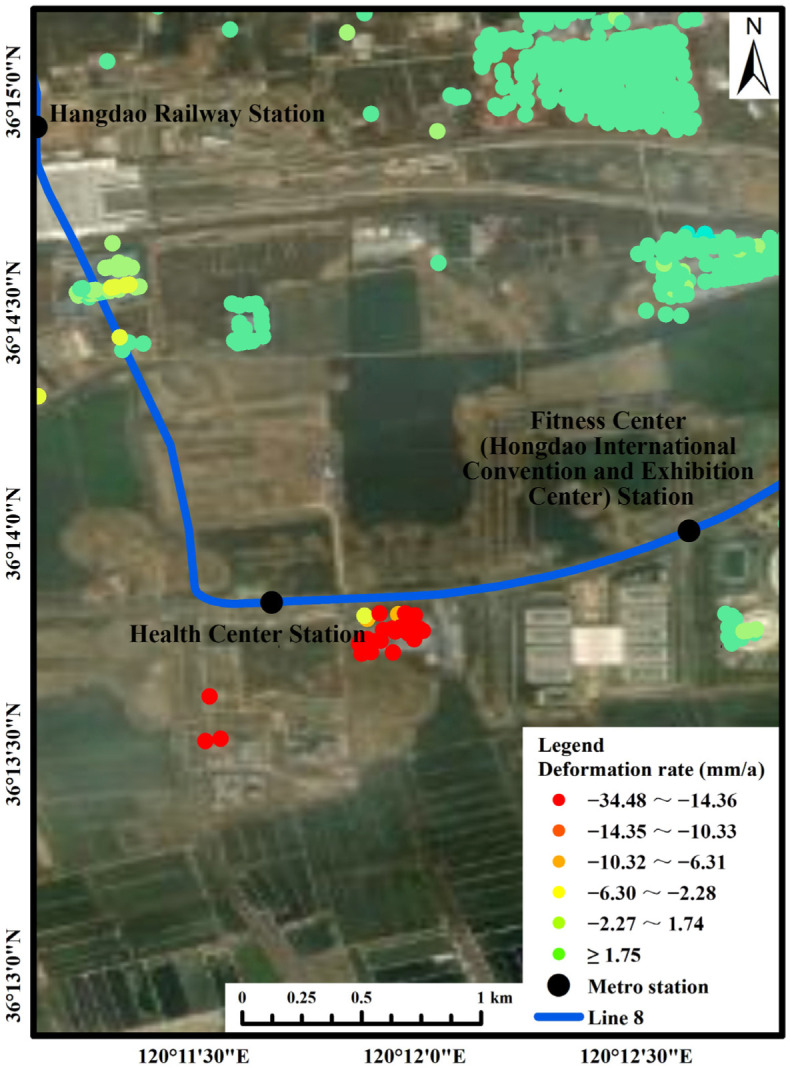
Deformation rate in Area A.

**Figure 11:**
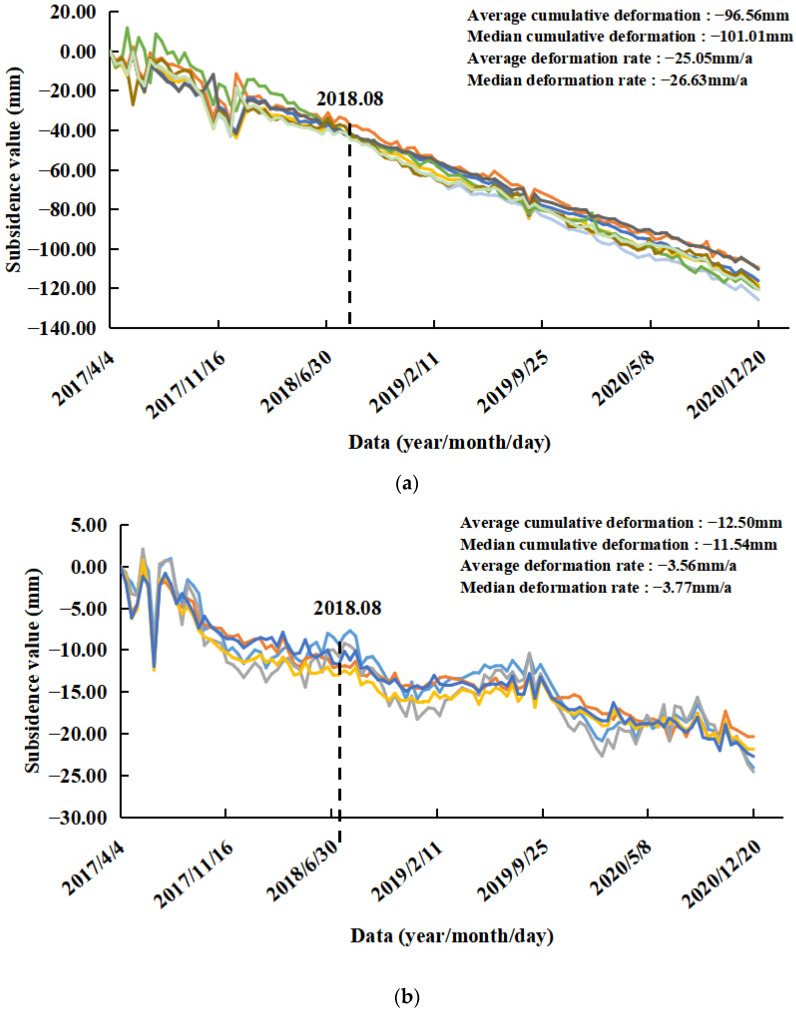
Part of the PS points subsidence: (**a**) the area to the south of the Health Center Station and (**b**) the area along the subway line from the Hongdao Railway Station to the Health Center Station. (Different colors represent different PS points).

In this study, a comprehensive analysis of Area A and Metro Line 8 was conducted (Figure 10). It can be seen from Figure 10 that the maximum subsidence rate on the south side of the Health Center Station reached 34.48 mm/a. From the deformation time of the PS point (Figure 9a), it can be seen that there was a sudden rebound phenomenon of 24.40 mm from 6 March to 18 March 2018. There was a concentrated subsidence area between the routes from the Hongdao Railway Station to the Health Center Station, where the average subsidence rate reached 3.84 mm/a. The site was divided into two time periods: before and after completion. Time period 1 was from April 2017 to August 2018, and time period 2 was from August 2018 to December 2020. The maximum cumulative subsidence in the construction period (time period 1) reached 42.30 mm, and the corresponding subsidence rate reached 31.73 mm/a. The maximum cumulative subsidence after the completion (time period 2) reached 84.68 mm, and the corresponding subsidence rate reached 35.04 mm/a. Combined with the construction time nodes, the part of the PS points subsidence of the area to the south of the Health Center Station and the area along the subway line from the Hongdao Railway Station to the Health Center Station was statistically analyzed (Figure 11). The overall subsidence trend of the south side of the Health Center Station was consistent and stable, but the land subsidence trend was more obvious. The average deformation rate was −25.05 mm/a, and the average cumulative deformation was −96.56 mm. The subsidence trend along Hongdao Railway Station to the Health Center Station was slow but unstable. The average deformation rate was −3.56 mm/a, and the average cumulative deformation was −12.50 mm. During time period 1, ground deformation was unstable in both study areas due to subway station construction. During time period 2, after the completion of the subway station, the ground shows a steady downward trend year by year. During the excavation and construction of the railway station, there was a great deal of concentrated pumping, which lowered the groundwater level. The line is located under the national highway, which supports a great deal of vehicle traffic. The above and other reasons combined to cause the current subsidence phenomenon. The analysis revealed that there was a serious land subsidence phenomenon in this area both during construction and after completion. The subsidence after construction was even more serious than that during construction.

**Figure 12:**
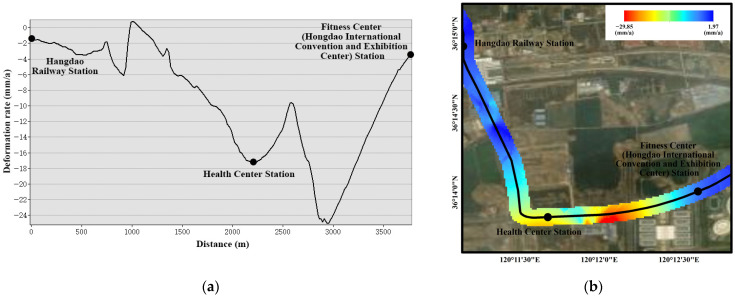
The subsidence of Hongdao Railway Station-Health Center Station-Fitness Center (Hongdao International Convention and Exhibition Center) Station: (**a**) the profile and (**b**) the buffer.

Because the subway belongs to a linear project, linear analysis can be carried out on it. According to the deformation rate of PS points on both sides of the subway line, the kriging interpolation method can be used to draw the raster layer, with the deformation rate as the z value. The deformation rate curve along the subway line direction was obtained through 3D profile analysis (Figure 12a). In addition, the subway center line was shifted 100 m to both sides as a buffer zone, and the subsidence around the subway can be intuitively seen (Figure 12b). As can be seen from the figures, the deformation rate of Health Center Station has exceeded −17 mm/a. In this section of the route, the subsidence of the central area from the Health Center Station to the Fitness Center (Hongdao International Convention and Exhibition Center) Station was the most serious. The conclusion is consistent with the previous analysis. Therefore, now that the normal operation of Metro Line 8 has begun, it is even more necessary to monitor the subway line along the Hongdao Railway Station-Health Center Station-Fitness Center (Hongdao International Convention and Exhibition Center) Station in real time and to implement corresponding measures to prevent various ground safety problems caused by subsidence.

**Figure 13:**
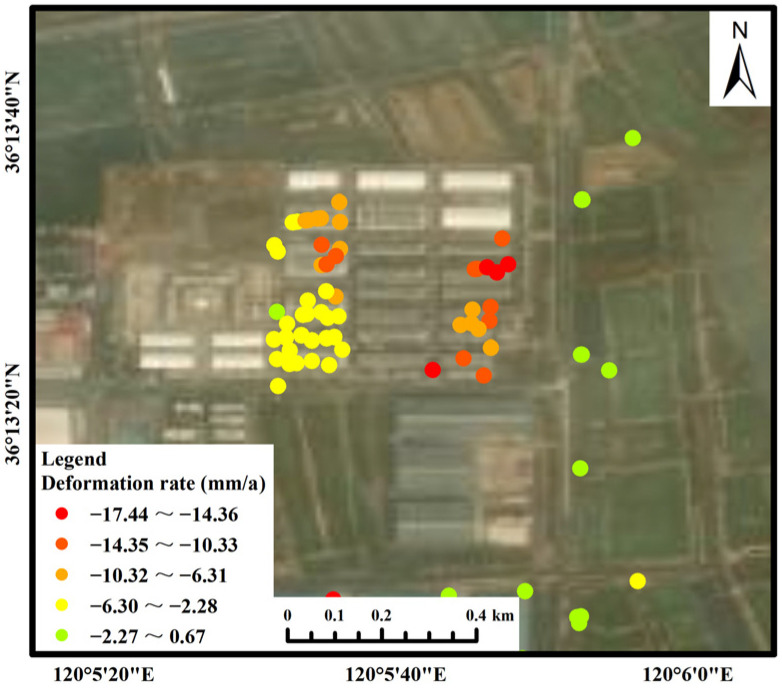
Deformation rate of a logistics company in Area B.

**Figure 14:**
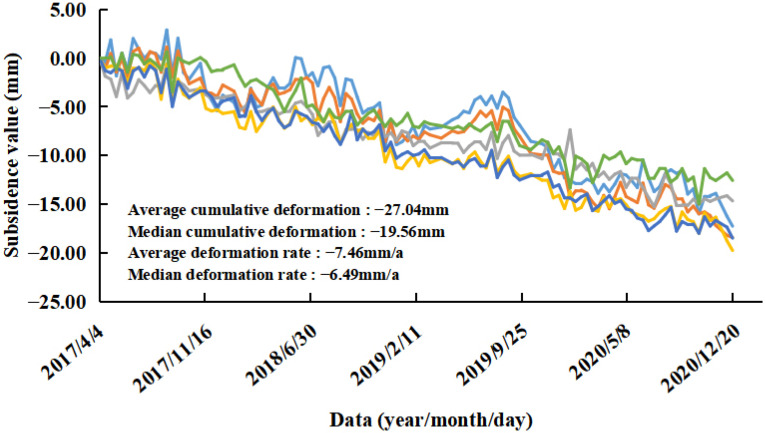
Part of the PS points subsidence of a logistics company in Area B. (Different colors represent different PS points).

Area B: This area included reservoirs/ponds, saline-alkali land, bare rock and gravel, and towns. The PS points were mainly located on buildings. The maximum subsidence rate in this area reached 28.44 mm/a, and the maximum cumulative subsidence reached 103.55 mm. From the deformation time of the corresponding PS point (Figure 9b), it can be seen that the PS point exhibited a slow downward trend overall, but sudden drops occurred during four periods: from 13 October to 6 November 2017; from 20 October to 1 November 2018; from 15 October to 8 November 2019; and from 21 October to 2 November 2020. It was found that each sudden drop period occurred from mid-October to early November and that there was a small range of rebound after the sudden drop. A concentrated subsidence area was identified in Area B, which was identified as a large logistics company. The subsidence is shown in Figure 13. The maximum subsidence rate in this area reached 17.44 mm/a, and the maximum cumulative subsidence reached 72.80 mm. For quantitative analysis, the part of the PS points subsidence of a logistics company in Area B was statistically analyzed (Figure 14). The overall subsidence trend of the area was consistent and stable, but some PS points had a more obvious subsidence trend than other PS points from 2019 to 2020. The PS points with obvious subsidence above were distributed in the east of the logistics company. The average deformation rate was −7.46 mm/a, and the average cumulative deformation was −27.04 mm.

**Figure 15:**
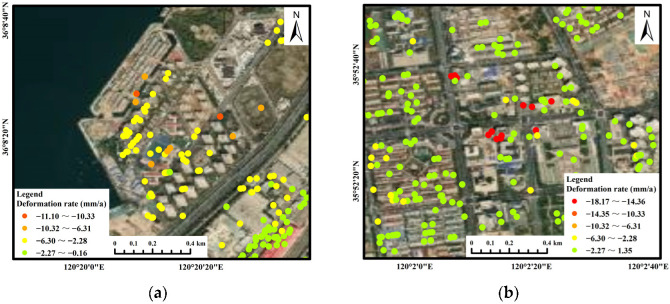
Deformation rates in Areas C and E: (**a**) Area C; and (**b**) Area E.

**Figure 16:**
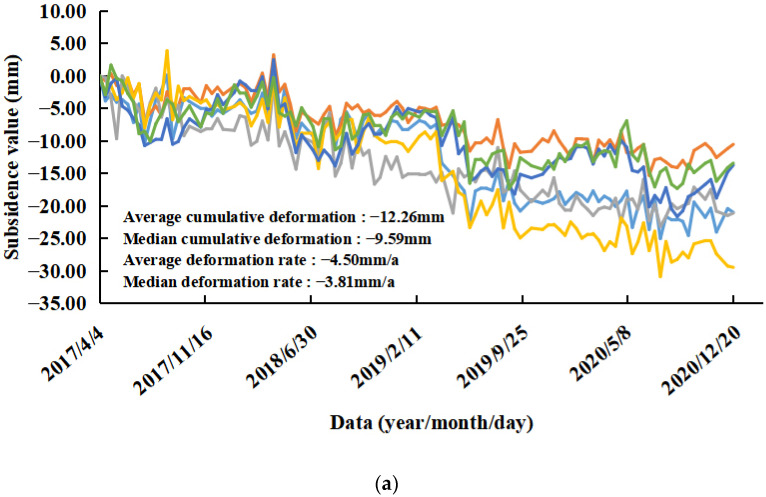

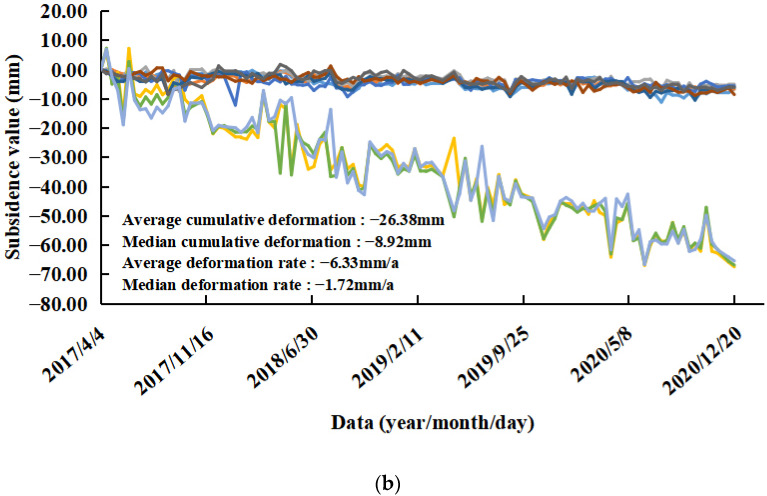
Part of the PS points subsidence of the concentrated subsidence area in Areas C and E: (**a**) Area C and (**b**) Area E. (Different colors represent different PS points).

Areas C and E: These areas are located in a town containing many high-rise buildings. This type of subsidence is caused by the presence of typical high-rise buildings. The PS points were located on buildings. The maximum subsidence rate in Area C reached 11.10 mm/a, and the maximum cumulative subsidence reached 36.87 mm. From the deformation time of the corresponding PS point (Figure 9c), it can be seen that the subsidence was obvious and that the cumulative deformation reached −19.09 mm from 25 March to 22 June 2019. The subsidence in Area C was concentrated in the coastal high-rise residential areas (Figure 15a). Statistical analysis was conducted on the part of the PS points in the concentrated subsidence area in Area C (Figure 16a). The subsidence trend of the whole area was consistent. The average deformation rate in the concentrated subsidence area was −4.50 mm/a, and the average cumulative deformation was −12.26 mm. The maximum subsidence rate in Area E reached 18.17 mm/a, and the maximum cumulative subsidence reached 75.38 mm. The subsidence in Area E was concentrated in the high-rise residential areas and business office buildings (Figure 15b). Statistical analysis was conducted on the part of the PS points in the concentrated subsidence area in Area E (Figure 16b). There were two trends in this area: one was the steady state of the ground, the other was the gradual subsidence trend, and the trend was very obvious. This phenomenon shows that only individual buildings in the area had subsidence and that the uneven subsidence of buildings cannot be excluded. Follow-up attention and monitoring are required for individual buildings. The average deformation rate in the concentrated subsidence area was −6.33 mm/a, and the average cumulative deformation was −26.38 mm.

Area D mainly included a port. The maximum subsidence rate in this area reached 32.16 mm/a, and the maximum cumulative subsidence reached 126.10 mm. From the deformation time of the corresponding PS point (Figure 9d), it can be seen that the PS point exhibited a slow downward trend overall, but there were three sudden drop periods: from 17 May to 29 May 2018; from 12 May to 24 May 2019; and from 18 May to 30 May 2020. It was found that each sudden drop period occurred in May and that there was a small range of rebound after the sudden drop. Area F mainly included reservoirs and ponds. The maximum subsidence rate in this area reached 11.90 mm/a, and the maximum cumulative subsidence reached 39.90 mm. The subsidence rates of the PS points in this area were mostly around 6.00 mm/a. Although the subsidence was not particularly obvious in this area, it is still necessary to continuously monitor the ground deformation state of the reservoirs and ponds. Area G mainly contained grasslands and fields. This area exhibited a slow subsidence trend overall. The maximum subsidence rate reached 11.29 mm/a, the maximum cumulative subsidence reached 33.41 mm, the average deformation rate was −2.14 mm/a, and the average cumulative deformation was −4.56 mm. From the deformation time of the corresponding PS point (Figure 9g), it can be seen that the deformation fluctuated greatly between 24 May and 9 September 2019. Area H mainly included towns, grasslands, and fields. This area also exhibited a slow subsidence trend overall. The maximum subsidence rate reached 32.31 mm/a, the maximum cumulative subsidence reached 121.67 mm, the average deformation rate was −0.98 mm/a, and the average cumulative deformation was −3.80 mm.

## 5. Discussion

### 5.1. Reliability Analysis

In order to verify the reliability of the research results of this study, the results of this study were compared and analyzed with the results of previous studies. The land deformation trend in the entire area in Qingdao is basically consistent with the results of Peng et al. [32], which shows that the land subsidence in the entire city was relatively stable. The presence of several typical subsidence areas along Jiaozhou Bay is basically consistent with the results of the study conducted by Peng et al. [32] in Area I. Among them, the most significant consistency between the results of this study and those of Peng et al. [32] is that the position of the maximum subsidence point remained consistent. In Reference [32], the cumulative subsidence of the feature point in Jiaozhou Bay from April 2017 to May 2019 was about 100 mm. In this study, the cumulative subsidence of PS points corresponding to the feature point in Jiaozhou Bay was about 80 mm. Although the results are a little smaller than the previous study, the overall subsidence trend is basically the same. In addition, it was found that the subsidence trend in the small area along Metro Line 13 (Jialingjiang road station-Xiangjiang road station) is also consistent with the results of Tao et al. [27]. The monitoring results of leveling points from 1 January 2018 to 30 November 2019 in Reference [27] were roughly compared with the results of PS points at the corresponding position in this study, and the difference was maintained between −4 mm and 2 mm. The above comparative analysis demonstrates the reliability of the results of this study.

### 5.2. Analysis of the Causes of Subsidence

In recent years, Qingdao has faced a new situation—i.e., rapid urban development and population expansion. The most obvious change was the continuous construction of the subway system. The subway construction has largely solved the crisis of living space and alleviated the current urban syndrome and other issues and has huge economic and social benefits, but it has several effects on the living environment. In the process of subway tunnel excavation, it is necessary to extract a large amount of groundwater, which causes a drop in the water level and the loss of soil layer. The additional stress induced by this process results in the compression of each stratum. The uneven nature of the subsidence is caused by the different degrees of compression in different places. Moreover, the redistribution of the stress and the concentration of the high stress result in deformation of the surrounding rock, the overlying strata, and the surface. During the operation of a subway tunnel, the long-term repeated action of the train’s vibration load may cause vibration subsidence and softening of the soil layer under the tunnel, resulting in plastic subsidence. A typical example of this type of subsidence is that in Area A. The land subsidence caused by the subway will cause varying degrees of damage to buildings/structures, underground pipelines, and other facilities along the line, and in serious cases, it may cause collapses and other accidents.

The emergence of high-rise buildings is another significant sign of rapid urban development. There are two main reasons for the subsidence of high-rise buildings. First, natural conditions have changed—that is, the engineering geology and hydrogeology have changed significantly. For example, the groundwater around high-rise buildings is excessively extracted, resulting in a significant decline in the water level, which leads to land subsidence. The second is the influence of the structure, load, and dynamic load of the building itself. The load of a high-rise building is very large and is borne by the foundation. The excessive bearing of the foundation will cause compression of the soil layer, which is manifested as building subsidence. Areas C and E are typical examples of this type of subsidence. When the uneven subsidence is serious, it will cause deformation of the buildings, which makes it difficult to ensure the safety of the buildings.

According to the results of the land subsidence monitoring in Qingdao from 2017 to 2020, the obvious subsidence areas in Qingdao were mainly concentrated in the coastal areas of Jiaozhou Bay and the Yellow Sea. The analysis revealed that the land subsidence in the above areas was mainly caused by human activities and excessive exploitation of groundwater. In the long term, land subsidence will become a bottleneck restricting urban construction and environmental protection [33,34]. When building height is less than 100 m, the subsidence critical value is 400 mm; when the height of a building is 100 m to 200 m, the critical subsidence value is 300 mm; when building height is 200 m to 250 m, the critical subsidence value is 200 mm [35]. Therefore, relevant departments need to pay attention to the specific situation of the local subsidence area, carry out reasonable control of it, and strengthen groundwater monitoring. When the subsidence phenomenon exceeds the regulation of groundwater use, it is necessary to adjust the mining level or stop mining. In serious cases, artificial recharge of groundwater can be adopted to promote the rise in water level and surface. For the subway safety system, the staff strictly abide by the engineering regulations during the construction period and take timely repair measures once the safety standards are exceeded. During the operation period, the subsidence area shall be further monitored in real time, and when the national safety regulations are exceeded, the operation shall be adjusted in time and measures shall be taken to improve the construction [1,2,3,17,18,19,20,21,22,23,24,25,32,36,37].

## 6. Conclusions

In this study, based on Sentinel-1A image, the land deformation in Qingdao City, China, from April 2017 to December 2020, was inverted using PS-InSAR technology. Combined with data for the local land types and subway construction, the distribution of the land subsidence in Qingdao City was analyzed in detail. The main conclusions of this study are as follows.

(1) From 2017 to 2020, the land subsidence in Qingdao was relatively stable. The deformation rate was mainly −34.48 to 5.77 mm/a, and the cumulative deformation was mainly −126.10 to 30.18 mm. The subsidence was mainly distributed in coastal areas (along the coasts of Jiaozhou Bay and the Yellow Sea) and inland areas (northeast Laixi City and central Pingdu City). The overall average deformation rate in each district/city was small, within 2.00 mm/a. The maximum subsidence rate occurred in the Chengyang District, and the deformation rate was −34.48 mm/a.
(2) The subsidence along the coasts of Jiaozhou Bay and the Yellow Sea was relatively scattered, exhibiting a sporadic distribution. According to the new findings of this study, the subsidence around the Health Center Station of Metro Line 8 was obvious, with a maximum subsidence rate of 34.48 mm/a. The maximum subsidence rate of a logistics company in Qingdao was 17.44 mm/a. Several high-rise residential areas and business office buildings were also found to have experienced obvious subsidence, with the maximum subsidence rate exceeding 11.00 mm/a. The subsidence in these areas was serious, so further attention should be paid to the safety of buildings and the ground surface.
(3) The northeastern part of Laixi City and the central part of Pingdu City were found to be in a state of slow subsidence and exhibited a concentrated circular distribution. The average subsidence rate in the area was within 2.50 mm/a, and the average cumulative subsidence was within 5.00 mm. Although the subsidence in this area was not obvious, it is also necessary to pay attention to the subsequent deformation of the buildings in this area.
(4) There were obvious differences in subsidence trend and degree near the Health Center Station caused by subway construction. Unstable deformation of the ground was caused by tunnel excavation during subway construction, while a steady subsidence of the ground was maintained due to the gravity effect after the completion of the construction. Therefore, further attention should be paid to the subsidence of the area after the operation of the subway line.
(5) The subsidence of buildings in Area C and E was caused by the excessive exploitation of groundwater and the load imposed by the overlying strata on the surface. Further analysis found that there was uneven subsidence of buildings in Area E, which posed a great threat to the safety of local life and property, requiring the management department to take corresponding measures to prevent disasters.

Combined with the development of Qingdao, the reasons for the subsidence in the typical areas were analyzed in detail based on the results of this study. Therefore, to some extent, this study provides a reference and data support for the relevant departments to address the problem of land subsidence and to monitor the safety of the buildings in these areas.

## Figures and Tables

**Table 1:** Properties of the Sentinel-1A dataset.

Parameter	Sentinel-1A
Band	C
Product Type	SLC
Polarization	VV
Sensor Mode	IW
Orbit direction	Ascending
Spatial resolution (m)	20
No. of image	108
Data range	April 2017–December 2020

## Data Availability

Data available on request from the corresponding author.
